# Dehydration Tolerance in Epidemic versus Nonepidemic MRSA Demonstrated by Isothermal Microcalorimetry

**DOI:** 10.1128/spectrum.00615-22

**Published:** 2022-08-16

**Authors:** Valérie O. Baede, Mehri Tavakol, Margreet C. Vos, Gwenan M. Knight, Willem J. B. van Wamel

**Affiliations:** a Department of Medical Microbiology and Infectious Diseases, Erasmus MC University Medical Center Rotterdam, Rotterdam, the Netherlands; b Centre for Mathematical Modelling of Infectious Diseases, Infectious Disease Epidemiology, London School of Hygiene and Tropical Medicine, London, United Kingdom; University of Mississippi

**Keywords:** *Staphylococcus aureus*, desiccation, environmental survival, epidemiological success, transmission

## Abstract

Methicillin-resistant Staphylococcus aureus (MRSA) clusters are considered epidemic or nonepidemic based on their ability to spread effectively. Successful transmission could be influenced by dehydration tolerance. Current methods for determination of dehydration tolerance lack accuracy. Here, a climate-controlled *in vitro* dehydration assay using isothermal microcalorimetry (IMC) was developed and linked with mathematical modeling to determine survival of 44 epidemic versus 54 nonepidemic MRSA strains from France, the United Kingdom, and the Netherlands after 1 week of dehydration. For each MRSA strain, the growth parameters time to end of first growth phase (*tmax* [h]) and maximal exponential growth rate (*μ*_m_) were deduced from IMC data for 3 experimental replicates, 3 different starting inocula, and before and after dehydration. If the maximal exponential growth rate was within predefined margins (±36% of the mean), a linear relationship between *tmax* and starting inoculum could be utilized to predict log reduction after dehydration for individual strains. With these criteria, 1,330 of 1,764 heat flow curves (data sets) (75%) could be analyzed to calculate the post-dehydration inoculum size, and thus the log reduction due to dehydration, for 90 of 98 strains (92%). Overall reduction was ~1 log after 1 week. No difference in dehydration tolerance was found between the epidemic and nonepidemic strains. Log reduction was negatively correlated with starting inoculum, indicating better survival of higher inocula. This study presents a framework to quantify bacterial survival. MRSA strains showed great capacity to persist in the environment, irrespective of epidemiological success. This finding strengthens the need for effective surface cleaning to contain MRSA transmission.

**IMPORTANCE** Methicillin-resistant Staphylococcus aureus (MRSA) is a major cause of infections globally. While some MRSA clusters have spread worldwide, others are not able to disseminate successfully beyond certain regions despite frequent introduction. Dehydration tolerance facilitates transmission in hospital environments through enhanced survival on surfaces and fomites, potentially explaining differences in transmission success between MRSA clusters. Unfortunately, the currently available techniques to determine dehydration tolerance of cluster-forming bacteria like S. aureus are labor-intensive and unreliable due to their dependence on quantitative culturing. In this study, bacterial survival was assessed in a newly developed assay using isothermal microcalorimetry. With this technique, the effect of drying can be determined without the disadvantages of quantitative culturing. In combination with a newly developed mathematical algorithm, we determined dehydration tolerance of a large number of MRSA strains in a systematic, unbiased, and robust manner.

## INTRODUCTION

Shortly after the introduction of methicillin into clinical use, the first methicillin-resistant Staphylococcus aureus (MRSA) were reported (1). In contrast to the wide genetic variety of methicillin-susceptible S. aureus (MSSA), MRSA presents a more clonal epidemiology (2). Investigation of its evolutionary origin showed the emergence of MRSA as five phylogenetic distinct clones, belonging to multilocus sequence typing (MLST) clonal complexes (CC) 5, CC8, CC22, CC30, and CC45 (2). Decades later, these MRSA clusters are still dominating on a global scale (3). In certain regions, historically dominant clonal clusters have been replaced by newly emerged MRSA types. One example is the replacement of the healthcare-associated CC30 EMRSA-16 by CC22 EMRSA-15 in the United Kingdom (4). In the American community, USA300 clone (CC8) replaced USA400 (CC1) as the most prevalent community-acquired MRSA (5). These observations demonstrate the variety in MRSA transmission success. The underlying mechanisms causing these remarkable shifts in space and time are unknown.

A variety of factors could play a role in MRSA transmission success, such as genetic flexibility, interaction with the host microbiome, human behavior such as crowding, antibiotic pressure, local differences in infection prevention policies, and environmental survival. So far, attempts to explain the clonal epidemiology of MRSA have mainly focused on host-pathogen interactions, while the role of environmental survival has been largely overlooked (6). Nevertheless, MRSA has previously been cultured from a wide variety of surfaces and fomites in hospitals (7–10). Even after terminal cleaning practices with 500 ppm chlorine, viable MRSA or S. aureus was present as dry surface biofilms on surfaces in intensive care units (11, 12). S. aureus dry surface biofilms have also been found on various hospital items (13). This suggests a potentially important role for fomites in the spread of MRSA. In this transmission route, MRSA bacteria in bodily fluids are deposited and dehydrated on a surface, after which they can be acquired and establish themselves in a new human host. Transmission of S. aureus from *in vitro*-grown dry surface biofilms to hands and then to fomites has been demonstrated *in vitro* (14). This transmission route relies on the capability of MRSA to survive dehydration and regrow in a more hospitable environment. Hence, differences in dehydration tolerance may play a role in determining whether a strain of MRSA is successful in transmission.

The results from the few studies that have investigated the role of dehydration tolerance in epidemic versus nonepidemic S. aureus are ambiguous (15–22). Furthermore, in these studies, only local strain collections were considered, overall sample numbers were low, definitions of epidemiological success varied, and climate-controlled conditions were lacking. Most importantly, all these studies quantified bacterial survival by counting CFU on agar. Because S. aureus forms grape-like clusters during growth due to incomplete separation of the daughter cells following division, both a single bacterial cell as well as a cluster of more cells will lead to the formation of a single CFU (23). Therefore, quantification by counting CFU can largely underestimate the effect of dehydration if part of a cluster dies, or alternatively overestimate this effect if very large clusters are formed. Hence, this method of quantifying the numbers of surviving bacteria is not reliable. Also, shaking, vortexing, or sonication of samples is necessary to release dehydrated bacteria from any material onto which they were deposited. Due to poor release of the bacteria from this material, the effects of dehydration can be overestimated as well. To overcome these limitations, different techniques for bacterial quantification are needed.

Isothermal microcalorimetry (IMC) is a technique which requires no sample preparation such as sample extraction or chemical labeling. Additionally, inoculated materials can be inserted directly for measurement, without the need for bacterial detachment via vortexing or sonication. In IMC, the total heat production of all active metabolic processes in a biological sample is monitored in real-time (24, 25). All metabolic processes produce heat, either at low levels for basic metabolism or at higher levels in the case of growth or stress responses. Earlier IMC studies have shown a linear relationship between inoculum size and lag time, represented by time of detection, in a range of bacterial species, including Escherichia coli, Pseudomonas putida, S. epidermidis, Proteus mirabilis, Lactobacillus reuter, and L. plantarum (24–28), but not, to our knowledge, for S. aureus, although IMC studies have explored S. aureus growth (29, 30). This linear relationship can be used to predict the size of the bacterial population based on the time of growth detection.

In this study, we describe the validation of IMC to capture S. aureus growth dynamics and its application to measure the survival of bacteria after dehydration. For this purpose, we combined an *in vitro* dehydration assay using IMC with mathematical modeling to predict bacterial survival after dehydration in a quantitative manner. This assay was used to investigate the contribution of dehydration tolerance to epidemiological success in a large representative collection of curated European MRSA strains collected by the MACOTRA study group.

## RESULTS

### Validation of IMC for *S. aureus* growth characterization and quantification.

**FIG 1:**
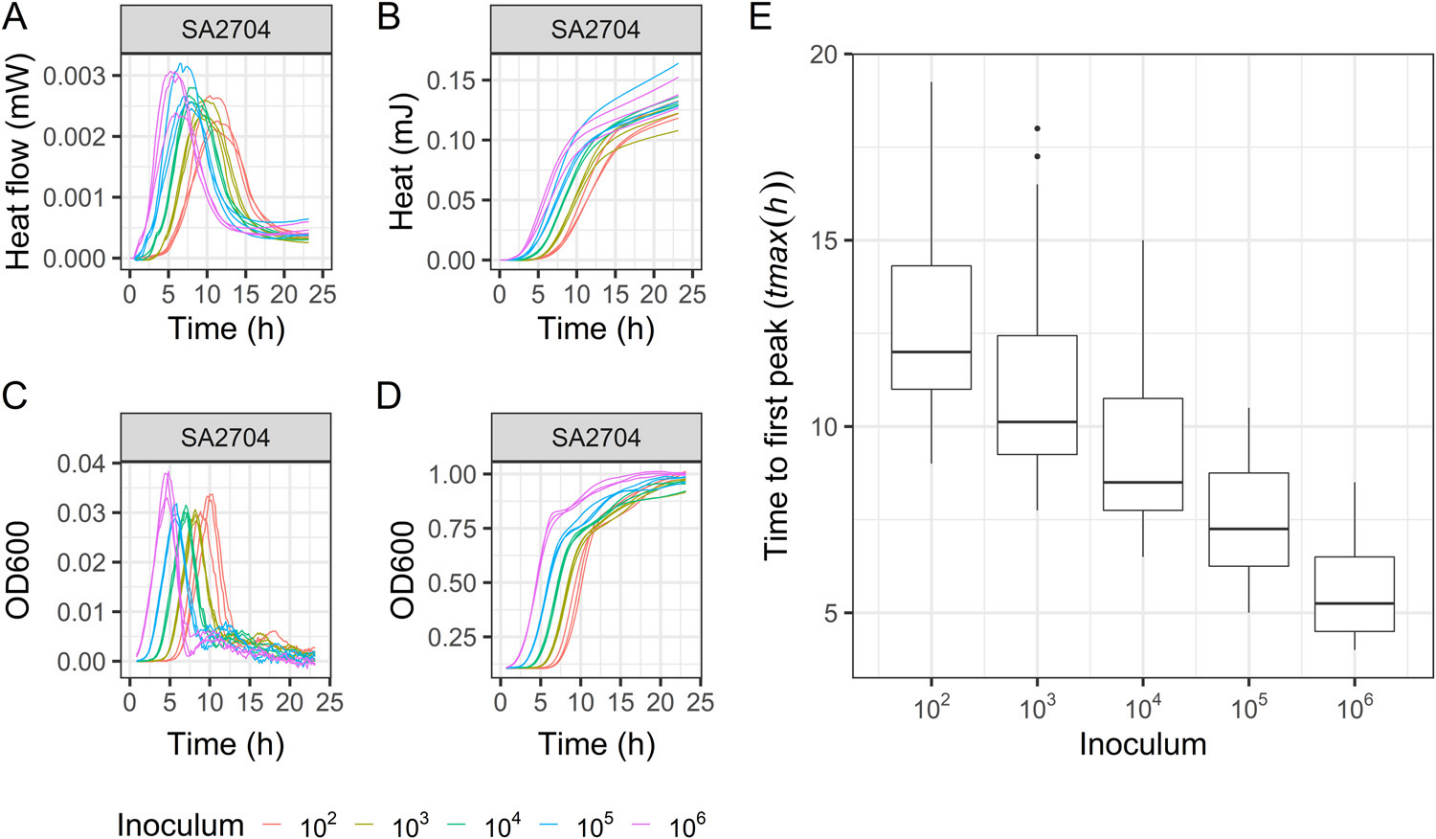
Validation of IMC for Staphylococcus aureus growth characterization. Growth curves were deduced from heat flow data (panel A: raw data; panel B: cumulated over time) and optical density data (panel C: differentiated between time steps; panel D: raw data) (example strain SA2704). The correlation between *tmax* from heat flow or optical density (OD) and inoculum size for strains SA2704, SAMUP15a, M116, and SAC042W was 0.97, 0.96, 0.96 and 0.95, respectively (based on 3 independent experiments, *P* < 0.001). Panel E: Linear relationship between inoculum and *tmax* for different starting inocula for 8 pilot methicillin-susceptible (MSSA) and methicillin-resistant S. aureus (MRSA) strains (see Supplemental data analysis section A for underlying data). Data are from 3 independent experiments.

For validation of IMC in S. aureus growth characterization, optical density (OD) growth curves and heat flow curves were compared in parallel experiments. Growth parameters were determined, and a strong correlation was found for the growth parameter *tmax* across the two data types (deduced as time to first peak in heat flow and time to maximum exponential growth in OD) for all four strains (*r* > 0.95) (Fig. 1). This indicates that heat flow curves obtained by IMC represent classical bacterial growth curves measured by OD under the study conditions, at least until the first peak in heat flow. Therefore, further data analysis was limited to the initial growth phase of the heat flow curve.

**FIG 2:**
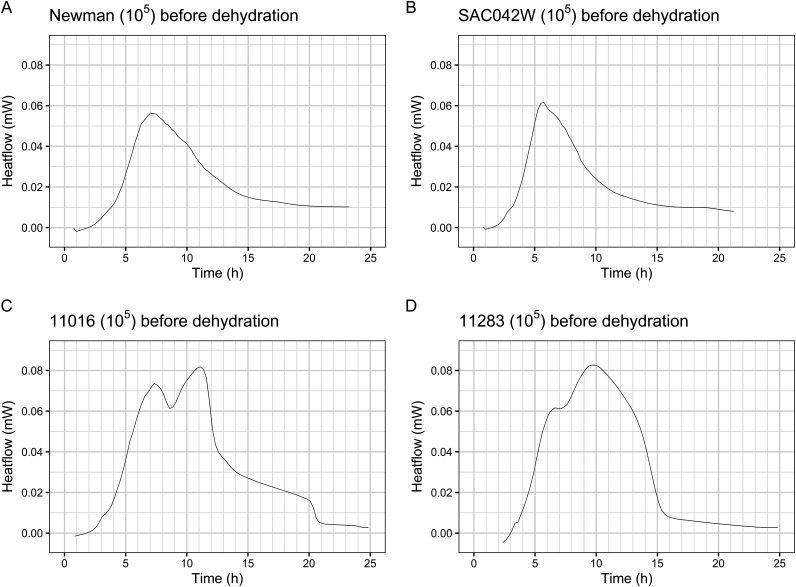
Heat flow profiles. Typically, S. aureus heat flow curves are characterized by a bell-like shape (examples in panels A and B). In 31 strains, multiple data sets with multiple heat flow peaks were observed (examples in panel C and D).

Next, heat flow curves were determined for 10-fold serial dilutions of 8 pilot MSSA and MRSA strains. Typically, S. aureus heat flow curves are characterized by a bell-like shape, usually reaching maximum heat flow within 5 h after lag time and declining to a stable heat flow level within a similar time period. Within this characteristic IMC profile, biological variation was seen, with each pilot strain displaying a unique kinetic fingerprint, shown by the strain-specific shape of obtained heat flow curves (examples shown in Fig. 2A and B). The observed profiles were comparable for different starting inocula of a strain, although occasionally decreased values for maximum heat flow and exponential growth rate were observed in lower starting inocula. However, we found an inoculum-dependent lag time, i.e., a longer lag time for lower starting inocula leading to a later heat flow peak. Based on these data, a linear relationship between inoculum size and *tmax* was confirmed for S. aureus (Fig. 1) as was seen earlier for other bacteria (24–26, 28).

**FIG 3:**
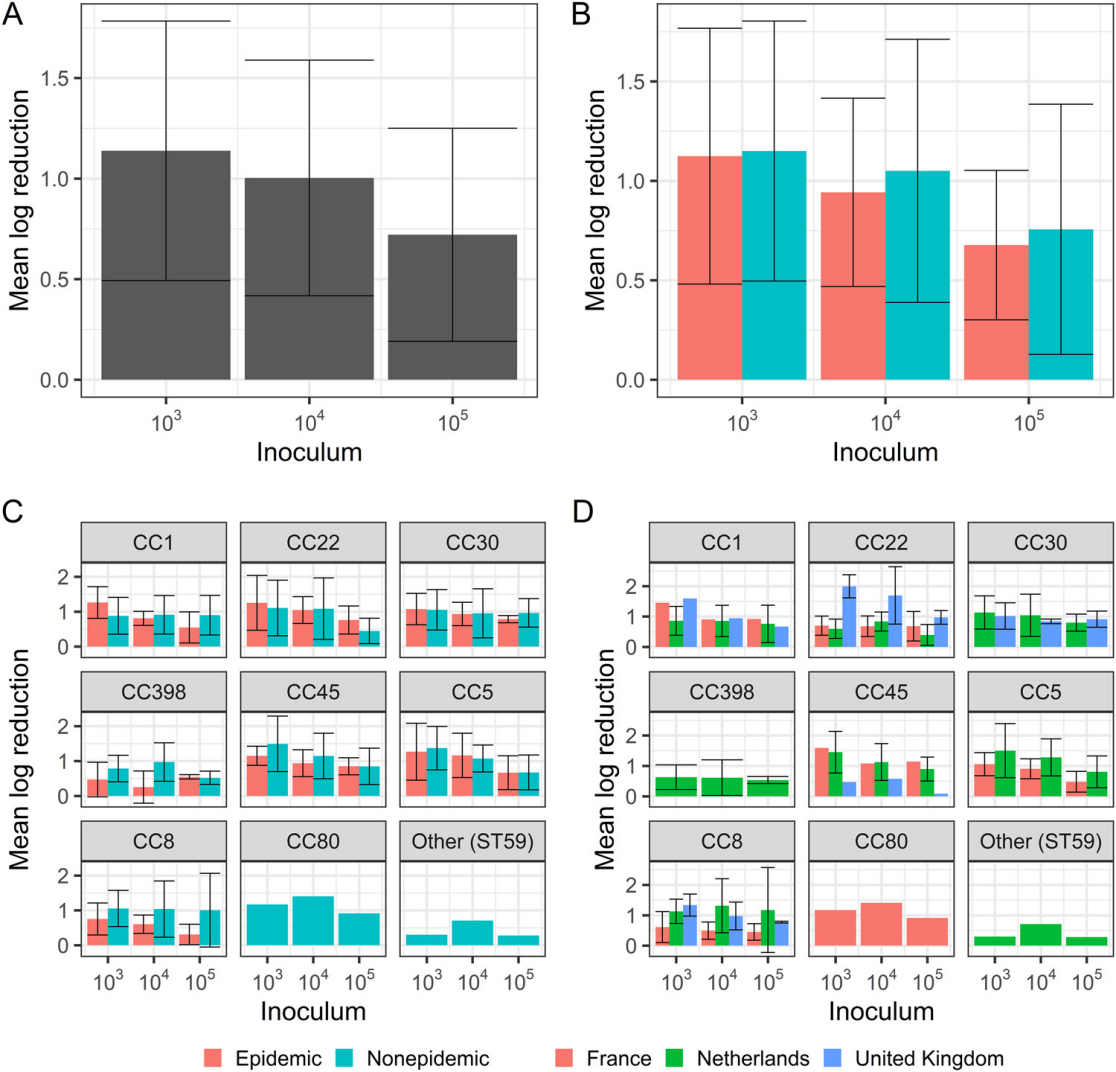
Mean log reduction results. Mean log reduction by (A) inoculum; (B) inoculum and success (color); (C) inoculum, success (color), and lineage (panel); and (D) inoculum, country (color), and lineage (panel). Bars indicate means with standard deviation error bars.

Together, these findings validated the use of IMC for S. aureus growth characterization and quantification.

### MRSA dehydration tolerance.

Heat flow curves were obtained for 98 MRSA strains before and after dehydration. Various steps of data cleaning were performed (Table S2), resulting in a final value of 1,330 data sets (75%) for 98 strains. Log reduction after dehydration could be determined for 90 strains (92%).

Overall, log reduction after 168 h of dehydration was 0.91 (standard deviation [SD] = 0.44). For epidemic strains, the mean log reduction over 168 h of dehydration was 0.92 (SD = 0.44). For nonepidemic strains, this was 0.95 (SD = 0.57). This difference was not significant (*t* = −0.34, *P* > 0.05). Log reduction varied significantly (F = 11.35, *P* < 0.001) by starting inoculum, from 1.14 (SD = 0.65) for 10^3^, to 1.00 (SD = 0.59) for 10^4^, to 0.72 (SD = 0.53) for 10^5^ (here, mean was taken over replicates and then per inoculum) (Fig. 3A). A *post hoc* Tukey’s multiple pairwise comparison test showed significant difference between 10^5^ and the two lower starting inocula (*P* < 0.05), but not between starting inocula 10^4^ and 10^3^. There was a similar trend in smaller reduction with higher inocula across epidemic and nonepidemic strains (Fig. 3B) and lineages (Fig. 3C). A trend toward higher dehydration tolerance was found for epidemic strains of CC8 (Fig. 3C). Furthermore, a trend toward lower dehydration tolerance was found for the UK CC22 strains compared to the French and Dutch CC22 isolates (Fig. 3D). Additional results on the linear relationship between inoculum and *tmax* for all strains, within-strain variation of log reduction, and the associations between log reduction, starting inoculum, epidemiological success, and country are given in Fig. S7, S8, and S9.

**FIG 4:**
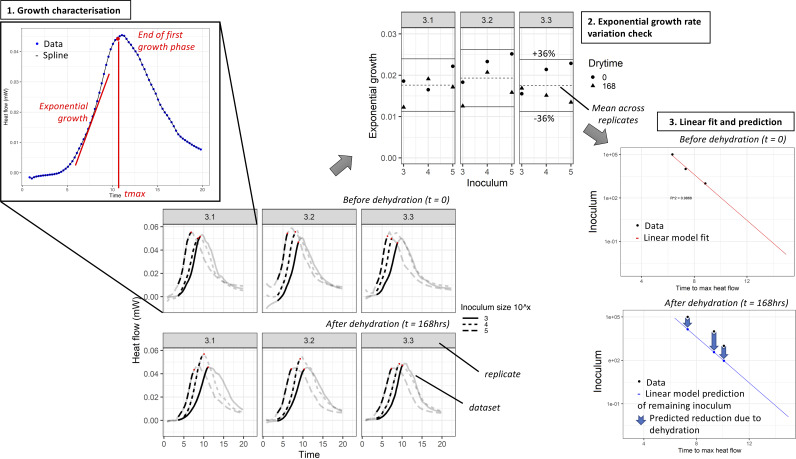
Schematic overview of all steps in data analysis. In the first step, smoothed cubic splines were fitted to the data and key growth parameters were extracted from the first growth phase of all data sets (e.g., *tmax* and maximal exponential growth rate, shown here in red). For step 2, data sets with ≤36% variability in maximal exponential growth rate were included for further analysis. In step 3, a linear model was fitted to *tmax* data prior to dehydration and the inoculum surviving dehydration was predicted based on the *tmax* after dehydration.

### Statistical model results.

In order to test for a difference in dehydration tolerance between epidemic and nonepidemic MRSA while accounting for starting inoculum, genetic lineage, and country of origin, a linear mixed-effects model was used. The effect size of epidemiological success was too small to explain differences in log reduction due to dehydration (b = −0.07, 95% confidence interval [CI] = −0.22 to 0.07, *P* = 0.31), i.e., no difference was found between the epidemic and nonepidemic MRSA strains. Differences in log reduction were explained by different starting inocula (*P* < 0.001), with an effect size of −0.21 (95% CI = −0.29 to −0.12).

## DISCUSSION

In this study, dehydration tolerance was explored for 98 MRSA strains with different epidemiological characteristics using a newly developed assay. No difference in dehydration tolerance was found between epidemic or nonepidemic strains of MRSA. Overall, we observed an average reduction of only approximately 1-log bacteria after 7 days of dehydration, indicating that dehydration tolerance is a common characteristic in S. aureus.

Interestingly, we found that MRSA survival was greater in higher starting inocula, indicating a bacterial density effect on survival. Chaibenjawong et al. (31) have demonstrated initial cell density-dependency in desiccation tolerance for S. aureus lab strain SH1000. To our knowledge, our study is the first to show this for multiple clinical strains of S. aureus and MRSA. This finding implies greater survival of bacteria, and thus possibility for transmission, in bodily fluids with a high bacterial load, such as pus. As a consequence, cleaning and disinfection protocols should be carefully implemented to ensure adequate removal of infected body fluids from the hospital environment after contamination. Equally important is compliance with hand hygiene protocols for health care workers to prevent transmission through fomites.

Earlier studies investigating dehydration tolerance of MRSA in epidemic versus nonepidemic strains presented ambiguous conclusions. After 15 days of dehydration on cotton swatches, log reduction ranged between 0.2 and 1.8, which was comparable to our findings (15). The same study showed that most epidemic strains lacked significant viability loss due to dehydration, but others were susceptible (15). Beard-Pegler et al. (17) found lower death rates for general epidemic strains compared to local epidemic strains after 7 days dehydration. Another study found that 2 MRSA outbreak strains survived longer and in higher quantities than 3 sporadic MRSA strains, although all strains survived at least for 225 days (19). In contrast, Farrington et al. (16) found lower dehydration tolerance in MRSA outbreak isolates compared to isolates from the hospital environment.

We found that dehydration tolerance was not significantly different between epidemic and nonepidemic MRSA strains. Our definition of epidemic versus nonepidemic strains, i.e., epidemiological success, was based on the relative prevalence of a genetic lineage within a country. For this, we depended on various surveillance programs, with different inclusion criteria and testing procedures, causing the definition of epidemiological success to vary per country and possibly affect our analyses. Also, we did not have enough statistical power to test differences in dehydration tolerance between genetic lineages. Nevertheless, our results show a trend toward high dehydration tolerance in epidemic strains of CC8. We could not confirm the findings of Knight et al. (21), which showed higher survival rates of epidemic CC22 strains after desiccation than strains of CC30, the clonal cluster it replaced as the most dominant clone in the United Kingdom. In our study, UK strains of CC22 were less tolerant to dehydration than Dutch or French strains of CC22. Other studies showed higher dehydration tolerance of different clones in their specific situations. In Italy, epidemic clone ST22-IV had increased survival capabilities in various stress conditions, including dehydration, than the CC5-ST228-I clone which it replaced (20). In the USA, clinical, colonization, and environmental S. aureus isolates of ST5 had higher dehydration tolerance compared to less epidemic strains of other STs in the study setting (22).

Together, these findings suggest that higher dehydration tolerance might benefit clones in their adaptation to a local niche in a geographic setting. However, considering the overall high survival of all MRSA in our study, dehydration tolerance seems to be a universal trait of S. aureus contributing to the global success of MRSA.

We observed a wide range of heat flow profiles representing high biological variability across our data (shown in Fig. 2 and 3). To account for this, we performed the analysis within strains across inocula. Lower starting inocula showed lower values for maximum heat flow and exponential growth rate. Approximately one-third of strains showed substantial variability from expected strain metabolism (double peaks, wide peaks, shoulders) after the first growth phase (see example in Fig. 2C and D). This indicated alternative metabolic processes after initial growth, supporting our analysis being limited to the initial growth phase. In some cases, this odd behavior was absent after dehydration, potentially pointing toward repression of these processes due to a stress response or the survival of a subpopulation with different metabolic behavior. Because the underlying mechanisms were not in the scope of this study, these were not investigated further. Additional work is needed to explore the metabolic processes and regulatory mechanisms underlying these observations and their roles in S. aureus stress responses.

While most studies use classical CFU counts for quantification of bacterial survival, we developed a highly reproducible assay using IMC. With this technique, the heat produced by viable bacteria was measured for quantification. First, the use of IMC for characterization of S. aureus growth was validated by the strong correlation between growth parameters in the initial growth phase derived from IMC- and OD-generated growth curves. For the extraction of growth parameters, we used a model-free approach by fitting smoothed cubic splines to heat flow curves. In contrast to traditional growth models, this model-free approach allows for higher flexibility in dealing with biological variation (32, 33). Additionally, we confirmed a linear relationship between time to end of first growth phase (*tmax*) and starting inoculum for S. aureus with the requirement of a constant maximal exponential growth rate (*μ*_m_). Based on these findings, a framework to deal with the large number of data, combining an *in vitro* dehydration assay with mathematical modeling, was developed to characterize bacterial time series in order to predict bacterial survival after dehydration in a quantitative manner.

Our dehydration assay was equipped with a climate chamber, ensuring climate-controlled conditions throughout the study. We chose climate conditions representing an indoor hospital environment, which is most relevant for MRSA. Higher temperatures or lower levels of relative humidity would increase the dehydration rate, which may have induced altered stress responses and therefore larger differences between strains (34). Also, longer dehydration periods are expected to show larger differences between strains, as shown in earlier studies (15, 16, 19). Although we observed the ability of MRSA to survive after a month of dehydration under the tested conditions (data not shown), the chosen setup could successfully indicate differences between strains. Because IMC is a closed system, limited oxygen availability might have influenced bacterial growth. To use IMC measurements for analysis of bacterial growth, these measurements need to be validated in comparison to an open system such as OD measurements. In our study, we confirmed a high correlation for growth parameters between both methods, which validated our approach. In the hospital environment, rehydration fluids would range from spilled water to blood, pus, and other nutrient-rich bodily fluids. To ensure high reproducibility of our results, we chose to use a nutrient-rich rehydration medium in this study. The high biological variability encountered in our data led to a series of data cleaning steps to maximize data usage for our study aim. The key assumption of linearity between inoculum and lag time required a consistent exponential growth rate across all data sets within a strain to be robust. Hence, we removed the most variable data sets (top 5%) and those for which this relationship was weak (low R^2^ value). This meant that we had high confidence in the predictive ability of the relationship for the final 75% of data sets that were included for the final data analysis, and we were able to determine log reduction due to dehydration for 90 of 98 clinical MRSA strains. The developed assay could be adapted to study the dehydration tolerance of other bacterial species that easily spread through the hospital environment, such as Enterococcus faecium and Acinetobacter baumanni. Additionally, IMC can be used to evaluate other stress-inducing conditions or monitor the real-time energy levels of biofilms or persisters throughout their development or treatment, since no disruptive sampling is needed.

To analyze our data, we developed an algorithm to extract characteristics of the first growth phase in peaked time series data, such as heat flow or the change per time step in OD. This flexible code can be used to extract similar growth characteristics for other data and allowed us to compare this large data set of growth curves in a systematic, unbiased manner. This was the result of much interdisciplinary discussion to develop an effective tool for this microbiological question and highlights the importance and potential of interdisciplinary research.

Overall, our results show the universal capability of S. aureus to survive dehydration in the environment with a small effect on viable numbers. Earlier studies have shown the persistence of MRSA on hospital items and surfaces in dry biofilms, even after terminal cleaning and disinfection (11–13, 35). This study helps by providing a well-explored open access method to quantify the growth and death of bacteria under a variety of circumstances. Together, these findings highlight the need for understanding survival and tolerance to environmental substances, including disinfectants.

## MATERIALS AND METHODS

### Strains.

In this study, a total of 98 MRSA strains from the MACOTRA strain collection were investigated, including 44 epidemic (successful) and 54 nonepidemic (unsuccessful) strains. The MACOTRA strain collection was compiled to study the factors explaining the clonal success of MRSA. Epidemic and nonepidemic strains were defined as those from a genetic lineage with a higher or lower relative prevalence within a country (36). A summary of included MACOTRA strains is given in Table 1 (see complete overview in Table S1). In addition, 8 well-studied MSSA and MRSA strains from different genetic backgrounds were included as pilot strains (Table 2) (37).

**TABLE 1:** MACOTRA strain characteristics^*a*^

Strain characteristic	Epidemic (*n*)	Nonepidemic (*n*)
Infection- or carriage-related		
Infection	26	30
Carriage	12	19
Unknown	6	5
Country		
France	10	10
Netherlands	24	35
United Kingdom	10	9
Year of isolation		
2006		1
2008	8	12
2009	6	3
2013		1
2014		5
2015	5	6
2016	2	4
2017	18	18
2018	5	4
MLST-CC		
CC1	3	5
CC5	10	9
CC8	5	10
CC22	14	12
CC30	6	4
CC45	4	10
CC80		1
CC398	2	2
Other (ST59)		1
Total	44	54

a MLST-CC, multilocus sequence typing clonal complex.

**TABLE 2:** Pilot strain characteristics^*a*^

Strain	Genetic background	Description	Reference
Newman	ST8	MSSA, laboratory strain	43
RN6390B	ST8	MSSA, laboratory strain	44
SA2704	ST72	MSSA, clinical isolate	45
MUP15a	CC15	MSSA, clinical isolate	46
M116	CC8, ST239	MRSA, clinical isolate	47
Mu50	CC5	MRSA, clinical VISA isolate	48
RWW146	CC398	MRSA	49, 50
SAC042W	CC8, USA300	MRSA, clinical isolate	51

a ST, sequence type; CC, clonal complex.

### Culture conditions.

Strains were cultured from frozen stock onto tryptic soy agar (TSA) supplemented with 5% sheep blood (Becton, Dickinson, Vianen, the Netherlands) at 37°C overnight. Bacterial suspensions were prepared at an OD at 600 nm (OD_600_) of 1.00 ± 0.05 (Ultrospec 10 Cell Density Meter; Amersham Biosciences, United Kingdom) in trypticase soy broth (TSB) (Becton, Dickinson, Vianen, the Netherlands) representing approximately 10^9^ CFU/mL. Subsequently, 10-fold serial dilutions were prepared in TSB in sterile U-bottom 96-well polystyrene (PS) microplates (Greiner Bio-One GmbH, Frickenhausen, Germany).

For classical growth curves, 10 μL of this logarithmic dilution series was added to a sterile U-bottom 96-well PS microplate filled with 190 μL TSB per well. Turbidity was measured every 10 min by OD_600_ in a microplate reader (Epoch 2, BioTek Instruments, VT) for at least 20 h. Before each measurement, the microplate was subjected to 1 min of double-orbital shaking at low speed.

### Climate-controlled dehydration assay using IMC.

Transparent polyvinyl chloride (PVC) strips (500 × 12 × 1 mm) (PR 107 4D; Bilcare Research GmbH, Staufen, Germany) were cut into coupons. After sterilization by autoclaving, coupons were inoculated with a 10-μL droplet of the logarithmic dilution series in duplicate. To determine reference heat flow before dehydration, one set of inoculated coupons were submerged into individual microcalorimeter vials filled with rehydration medium containing 290 μL TSB to reach a total volume of 300 μL, and placed in the isothermal microcalorimeter (calScreener, Symcel AB, Spånga, Sweden). Microcalorimeter vials were allowed a pre-incubation period of 30 min to reach thermal equilibrium at 37°C. Heat production of individual vials was measured as heat flow (μW) for at least 20 h. The other set of inoculated coupons was placed in a climate chamber (HPP110; Memmert GmbH + Co. KG, Büchenbach, Germany) for dehydration at 21°C, 40% relative humidity, representing an indoor environment. After dehydration for 168 h, coupons were placed in prepared microcalorimeter vials containing 290 μL TSB and 10 μL H_2_O (WFI for Cell Culture; Gibco, Bleiswijk, the Netherlands) and processed by IMC as previously described.

IMC is a highly sensitive technique, which leads to high data variability. To reduce technical variation, samples were handled following a robust protocol (details in Supplemental data analysis section B, data cleaning 1). Obtained heat flow curves were visually inspected for the occurrence of nontypical S. aureus heat flow patterns suggesting contamination. Potential contaminated vial contents were cultured to confirm bacterial contamination. In cases of contamination or technical issues, IMC data were excluded from further analysis (Supplemental data analysis section B, data cleaning 1). An external baseline based on a medium blank was used to correct all obtained heat flow curves to enable data comparison between separate IMC measurements (personal communication, Magnus Jansson; Symcel AB, Sweden). Data were exported at 900-s intervals. Data obtained within the first 3.5 h of measurement were excluded to guarantee thermal equilibrium (38).

### Data analysis.

**(i) Comparison of heat flow curves and OD growth curves.** Parallel experiments with the same bacterial dilution series of 4 pilot MSSA and MRSA strains were performed using OD and IMC. The obtained data were compared to determine whether IMC data could be used to quantify key characteristics of bacterial growth. Because heat flow in IMC is measured in real-time and collected for every time step, but bacterial density in OD is cumulated over time, the differences in OD data were calculated for each time step to enable the comparison of these data (Supplemental data analysis, section A). The R *grofit* and *tidyverse* packages were used to fit smoothed cubic splines to heat flow curves and differentiated OD growth curves (32, 39, 40). Growth parameter time to maximum heat flow in IMC or time to maximum exponential growth in OD was deduced. Spearman’s correlation coefficients were computed to compare obtained values from heat flow curves and differentiated OD curves using the *cor* function in R *stats* package (39). Data of three independent replicates were used for analysis.

**(ii) Extraction of growth characteristics.** For further analysis of peaked time series data such as IMC data, a new algorithm was written to extract key growth parameters from the first growth phase: time to end of first growth phase (*tmax* [h]) and maximal exponential growth rate (*μ*_m_) (Supplemental data analysis, section C). First, smoothed cubic splines were fitted to the heat flow curves (32, 39, 40). Next, the time to first peak was determined by characterizing peaks and shoulders (minor plateaus) in the data. The earliest point of the first plateau (either around a peak or shoulder) was set as the time to first peak (*tmax* [h]). When no plateau existed, the time to first peak was set as *tmax*. For the data up to *tmax*, a spline was fitted and the maximal exponential growth rate (*μ*_m_) was extracted.

**(iii) Heat flow analysis: prediction of log reduction.** For each of the 98 MRSA strains, up to 18 data sets of growth parameters were extracted from the heat flow data from 3 experimental replicates, 3 different starting inocula, before and after dehydration for 168 h. Because lag time scales linearly with inoculum size, *tmax* in heat flow is linearly correlated with inoculum size under the assumption of a constant maximum exponential growth rate (*μ*_m_). Thus, *μ*_m_ must be comparable across inocula and before and after dehydration within a replicate. This was assessed by comparing the *μ*_m_ from an individual data set to the mean *μ*_m_ over all data sets in a replicate. Next, the variation in *μ*_m_ across strains was explored. We chose to exclude a data set if its *μ*_m_ deviated by more than a chosen percentage cutoff from the mean *μ*_m_ across the replicate. A replicate was excluded if more than two of its data sets were excluded. A strain was excluded if it had only one replicate (of a possible three) remaining. The cutoff for acceptable variability was determined so it excluded the top 5% of strains with the greatest variability (Supplemental data analysis, section D, data cleaning 2). After removing these most variable strains, we used this cutoff to iteratively remove data sets: first, any data set with *μ*_m_ outside the cutoff from the mean was removed, then the mean *μ*_m_ was recalculated, etc.

For the remaining data, a linear model was fitted to the *tmax* data prior to dehydration (equation below) for each replicate to estimate replicate specific intercept (*a*) and gradient (*b*) values.

log(inoculum)=a + b×tmax

Using this parameterization, the inoculum which survived dehydration could be predicted within a replicate based on the *tmax* observed after dehydration. The difference between starting inoculum and viable inoculum after dehydration was defined as log reduction. Fig. 4 shows a schematic overview of data analysis. Only those replicates with a linear fit where R^2^ > 0.75 and for which two or more data sets were available prior to dehydration were used in further analysis (Supplemental data analysis, section E, data cleaning 3 and 4).

Unless otherwise stated, all values are reported as the mean for the strain, which is the mean over the replicates (up to three) of the mean log reduction over all inocula in a replicate. Fewer than three replicates would remain in the final analysis if data sets had been removed in any of the four data cleaning steps described above.

During all steps of data analysis, strain metadata were blinded for the executing researcher (G.M.K.).

### Statistical analysis.

For statistical analysis of log reduction, a linear mixed-effects model was built using the R *lme4* and *lmerTest* packages (41, 42). Epidemiological success and starting inoculum were taken as fixed effects. To account for selection bias, genetic lineage and originating country were taken as random effects.

### Data availability.

All strain metadata, OD and IMC data, and accompanying analysis codes are available from the GitHub repository (https://github.com/gwenknight/strain_growth). MRSA strains included in this study are available on request from the MACOTRA study group.
