# Supplementary material for: Dehydration Tolerance in Epidemic versus Nonepidemic MRSA Demonstrated by Isothermal Microcalorimetry
Source: Microbiol Spectr. 2022 Aug 16;10(5):e00615-22. doi: 10.1128/spectrum.00615-22 (PMC9602581; doi:10.1128/spectrum.00615-22)
Supplement: Supplemental file 1 — Supplemental material. Download spectrum.00615-22-s0001.pdf, PDF file, 2.1 MB [file spectrum.00615-22-s0001.pdf]

Supplemental data analysis

Section A: Validation

Comparison of Heat Flow and Optical Density data

CODE: proof_of_principle.R.

In order to validate use of IMC data, we compared heat flow and optical density data from the bacterial dilution series. As can be seen in Figure S1, there is a high level of similarity in the time series behavior, especially over the first 5-10 hours.

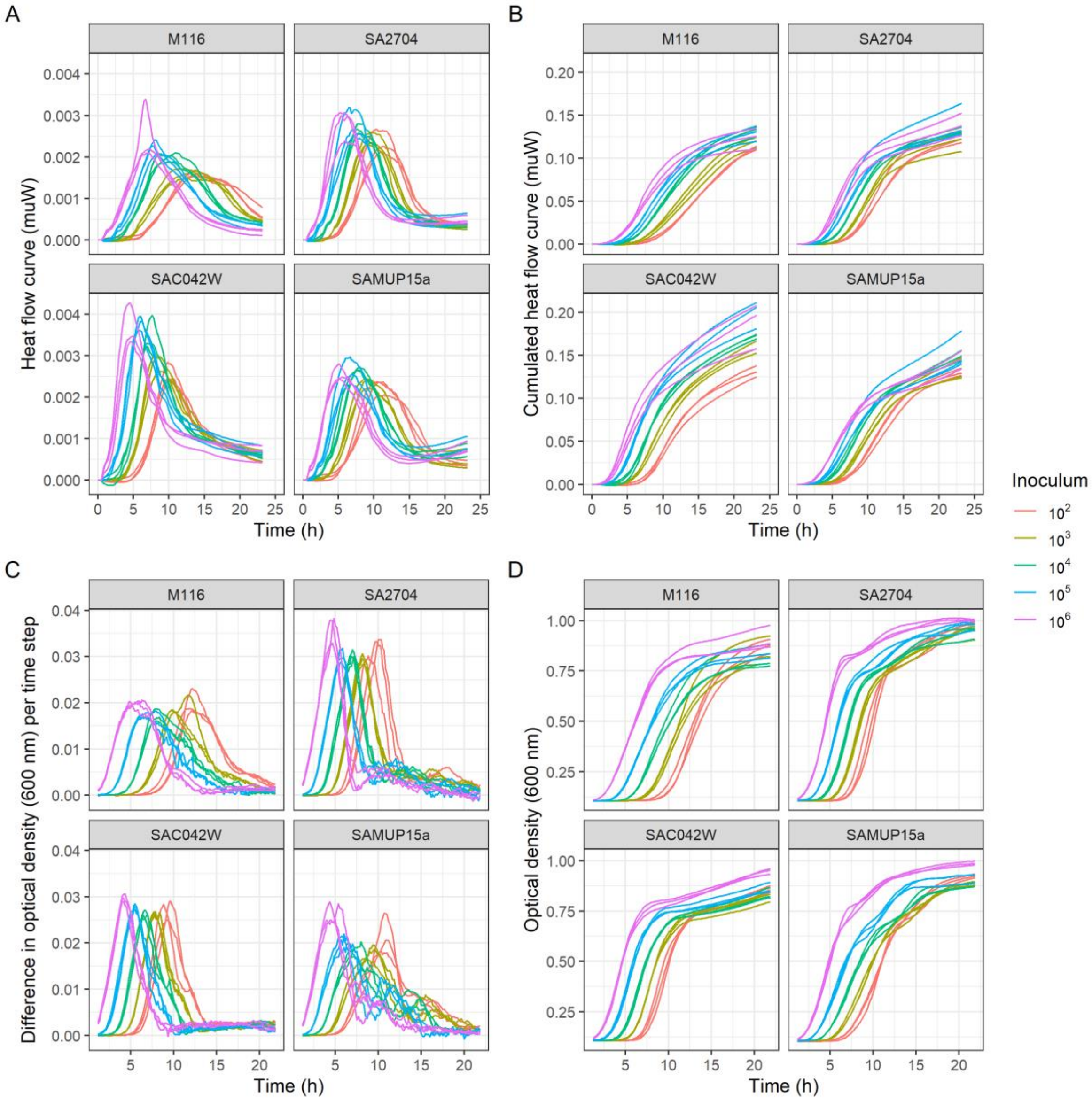

Figure S1. Comparison plots of heat flow data (raw (A) or cumulated over time (B)) and optical density data (raw (D) or the difference between time steps (C)).

The algorithm for growth data characteristic extraction as described in section C was written to work with time series data with a clear peak, as it was based on the heat flow data (as in Figure S1A). Hence, for the comparison of heat flow and optical density data the change in raw optical density data per time step was calculated. Effectively, OD measures the density of the bacterial culture reflecting the cumulation of bacterial growth, whilst heat flow curves are snapshots of heat flow at each time. Hence for a comparison, the difference between OD values at each time step was produced (Figure S1C). The peak in the differentiated optical density data therefore represents the time at which the bacterial exponential growth rate is maximal. For comparison, time to peak in heat flow and time to maximum exponential growth rate in optical density is given in figure S2.

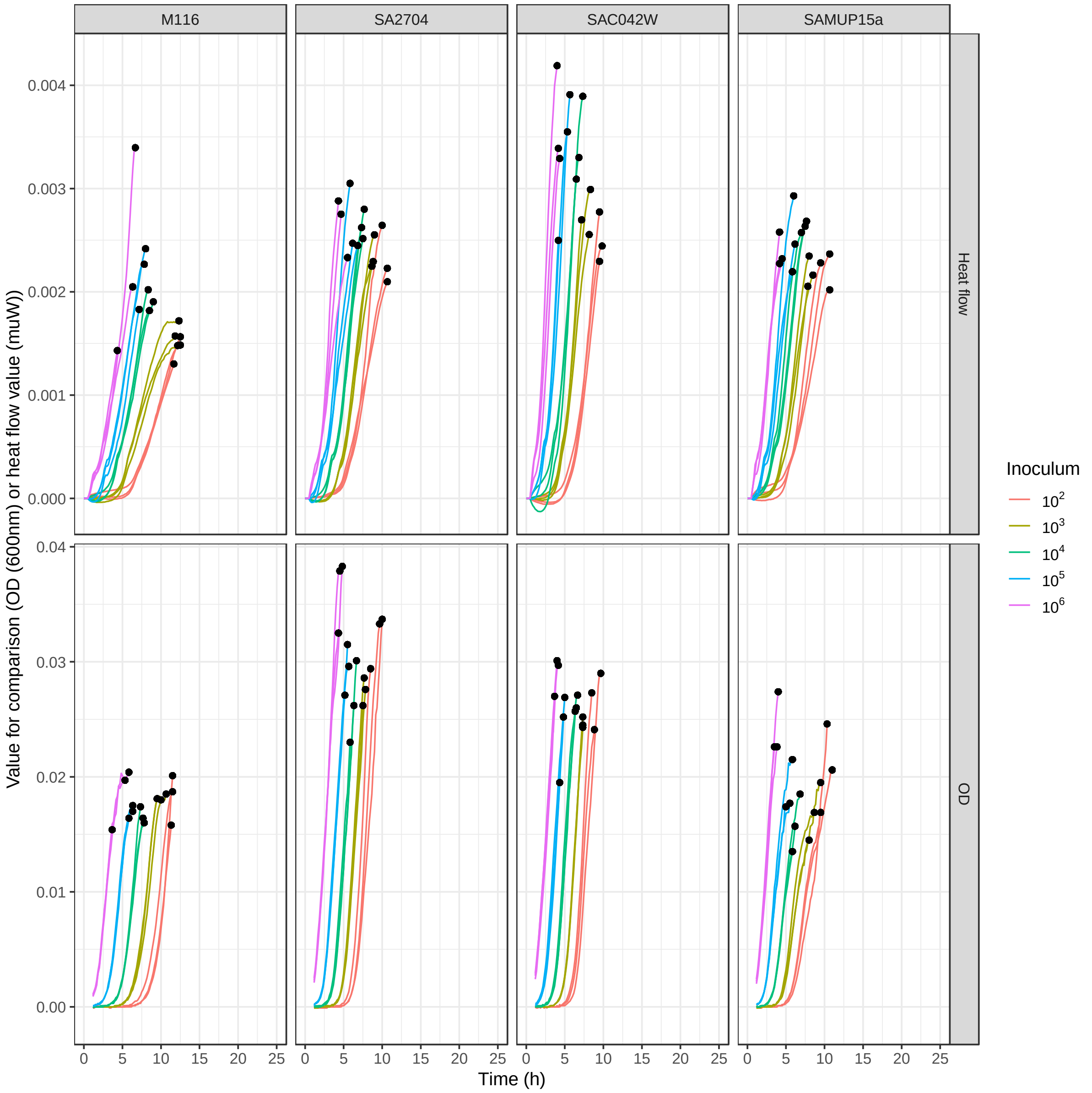

Figure S2. Time to first peak for heat flow data (top row) and optical density (OD) data (bottom row) for the four compared strains at each inoculum.

Linear relationship between inoculum and *tmax*

CODE: 2_analysis_non_macotra.R

As an initial check, we explored the relationship between inoculum size and time to first peak in heat flow. For the 8 pilot *S. aureus* strains in this analysis the results are shown in Figure S3.

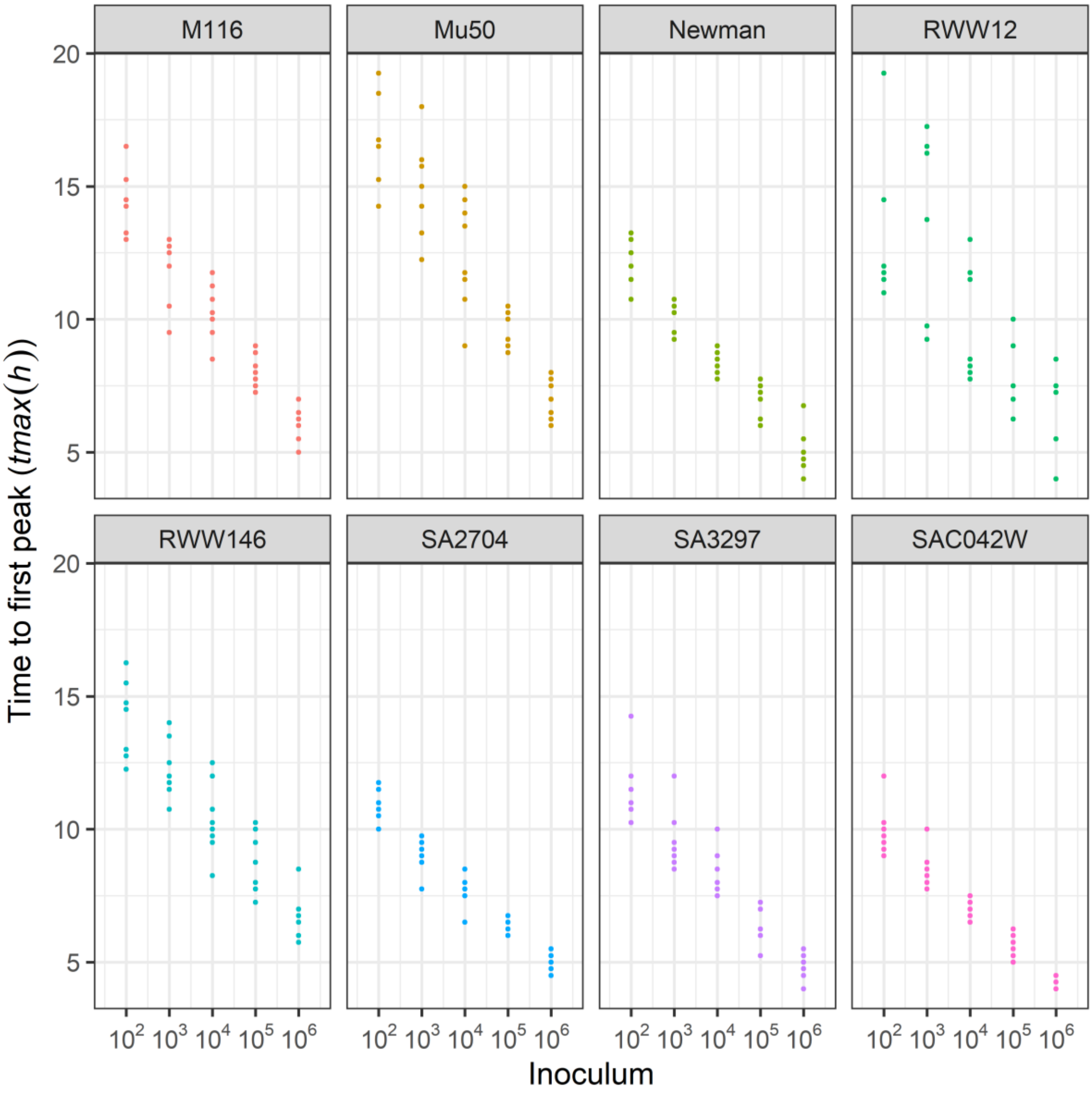

Figure S3. Time to first peak (*tmax* (h)) in heat flow against inoculum for each of the 8 pilot *S. aureus* strains

Section B – Labelling and data cleaning 1

IMC is a highly sensitive technique, which leads to high data variability. Technical variation was minimised as follows: Symcel provided all required maintenance to the used CalScreener, assuring accurate measurements. Prepared PVC coupons were placed directly into the calorimeter vials without the use of an insert, so there was no chance of condensation build-up between the insert and the vial. All samples were carefully pipetted to ensure no air bubbles were present that would distort our measurements. Leaking of vials due to incorrect closing of the vial lid is easily detected as this causes a constant distorted signal due to the leaking of heat. Potentially contamination of samples was detected by non-typical *S. aureus* heat flow patterns in combination with confirmed bacterial contamination through culturing. All technical issues were assessed by this data cleaning step.

For each original set of experimental data (corresponding to a single set of strains) 1 – 14, visual inspection revealed single datasets that should be removed. These were manually excluded from the data before use in the analysis. This visual inspection can be replicated with the corresponding removals detailed in the individual 1_data_set#.R files. These files also contain all labelling information for raw experimental data, including strain, inoculum and drytime information.

CODE: 1_data_set#.R files. Run 1_data_cleaning.R to clean all data.

The original data consists of
- 98 strains
- 3 inocula
- 3 replicates
- 2 time point measures (before dehydration and after 168h (7 days) of dehydration)

One time series of data for a replicate of a strain is called a dataset. Originally there were (98 x 3 x 3 x 2 =) 1764 datasets.

Section C – Time series analysis

An algorithm was written which would take any time series data (such as heat flow data over time) and extract the time to the end of the first growth phase. In essence, a smooth spline is fitted to the data and characteristics of the curve extracted.

CODE: 2_analysis.R Extracts the characteristics of the curve into a single matrix and adds the key parameters to the time series data.

This analysis uses the function cut_extract.R within functions_for_heat_curves.R

The steps within cut_extract.R are:
(1) Fit a smooth spline to the dataset
(2) Determine the maximum value
(3) Determine if the peak is broad and explore multiple peaks (defined to be a point greater than 5 points either side). The “odd” characteristics explore the behavior of the strain but do not affect the analysis here.
(4) Determine if there is a shoulder. This is a slowing down of growth and a mini-plateau before continuation of upwards growth.
(5) Determine if there is a double peak. This would happen if two normal curves could be fit to the data. The number of these is reported but not analyzed further here.

(6) Taking the above analysis, the dataset is cut at the shoulder or first peak to explore just the initial growth

(7) The peaks in this data are explored. If there is a peak, then this is reassigned as the new end of first growth (*tmax)*

(8) Then this data is explored to see if there is any earlier plateauing or a shoulder. This is done by fitting a linear model to segments of the data and reassigning the end of first growth (*tmax)* to a point when the gradient of these segments varies substantially.

(9) A smoothed spline is then fitted to this new data to give the maximum exponential growth rate (μ_{m}) of this first growth.

Section D – Analysis of maximum exponential growth rate variation, data cleaning 2 and log reduction calculation

Maximum exponential growth rate constant

What cutoff should be used to remove those strains with the greatest inter-replicate maximum exponential growth rate variation?

Method

CODE: 3_exponential_growth_variation.R	Explores and plots the output to determine maximum exponential growth rate variation

In order for the linear assumption between time to first peak *(tmax)* and inocula to hold, the maximum exponential growth rate must be constant across inocula and pre- and post-dehydration within a replicate. To explore the variability in maximum exponential growth rate, mean values were calculated across replicates within strains. A cutoff percentage from this mean was determined that removed those top 5% of strains that had the greatest variability in maximum exponential growth rate.

A dataset would be removed if its maximum exponential growth (μ_m) was outside of the mean +/- a percentage cutoff over the replicate. A replicate would be removed if more than a third of the datasets within a certain dry time (e.g. pre- or post-dehydration) would be removed. Normally there are three datasets, so if two or more are removed due to having a maximum exponential growth outside the range then the replicate has insufficient data at this dry time. Sometimes a dataset has already been removed (due to abnormal measurements, see data cleaning above) and if any other single dataset is removed for this replicate and dry time, then the replicate would be removed. Hence a replicate would be kept if it had at least two datasets per dry time, but removed if at any dry time there was only 1 dataset with a maximum exponential growth rate within a +/- percentage cutoff of the mean over the replicate.

Results

The exploration of variation in maximum exponential growth rate is shown in Figure S4. We decided to exclude those strains that have the greatest variability in maximum exponential growth: we chose initially to remove the highest 5% of variability and so set a percentage cutoff of within 36% of the mean (Figure 1C).

A summary of remaining data after filtering on maximum exponential growth rate is shown in Table S1.

Number of datasets	5	6	8	10	11	12	13	14	15	16	17	18
Number of strains	8	2	1	7	5	4	1	3	5	9	28	25

Table S1. *The remaining data for analysis after filtering on maximum exponential growth rate, with a total of 1433 datasets for 98 strains*

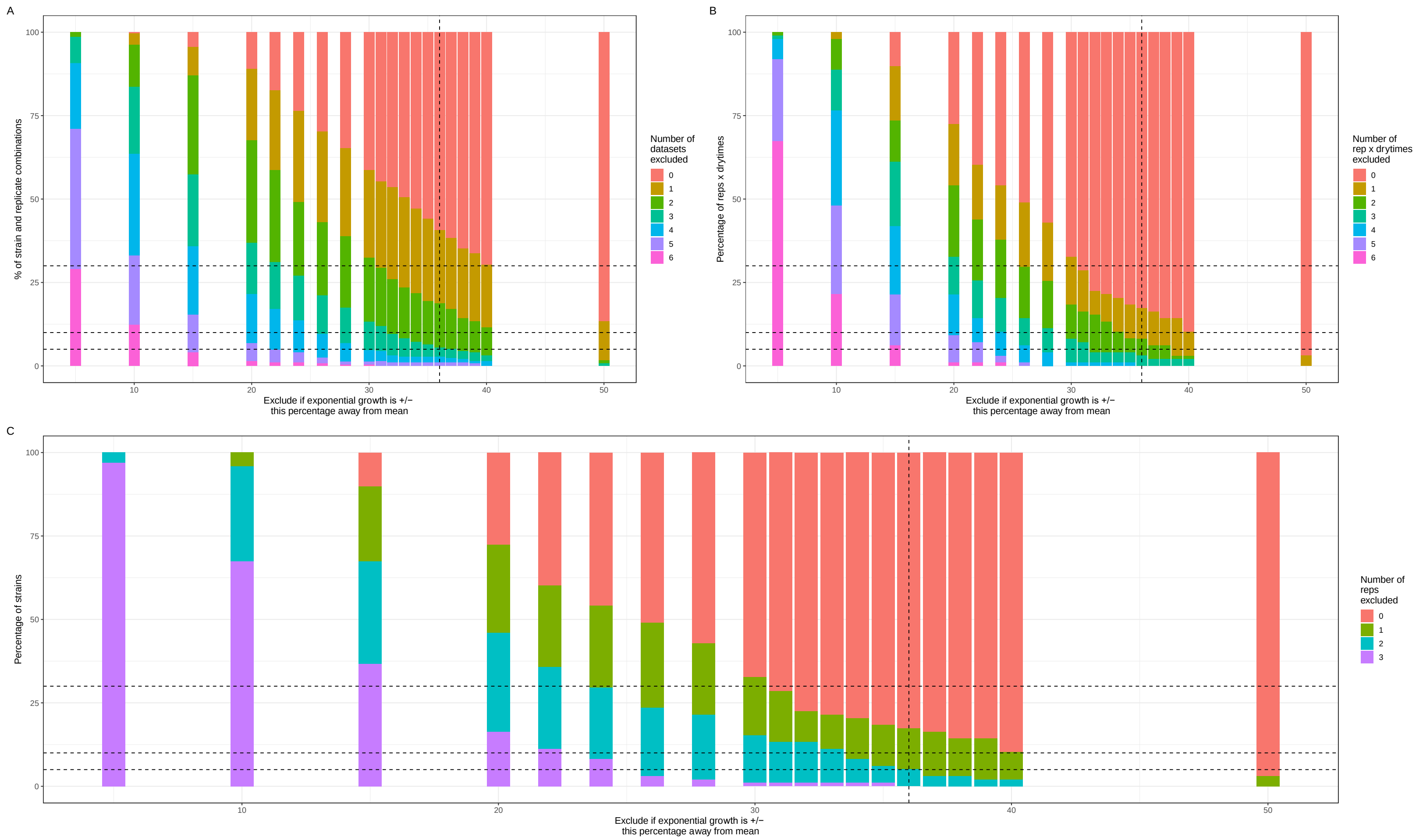

Figure S4. Exploration of maximum exponential growth rate variation. For all graphs, the x-axis is the percentage cutoff value from the mean for excluding the dataset. Then the y-axis varies in each graph. For (A) each color is the number of datasets excluded within a single replicate, for (B) it is within a strain how many replicate and drytimes would be excluded (e.g. replicate 3.1, no dehydration or replicate 3.2, 168h dehydration) and for (C) it is within a strain, how many replicates would be excluded. The horizontal lines represent a 5%, 10% and 30% cutoff for variability. The vertical line represents the final choice of 36% to remove the 5% of strains (in (C)) with the greatest variability.

Once the cutoff was determined, we removed datasets iteratively for each strain. To do this we

(1) Calculated the mean over a replicate
(2) Calculate the allowable range for maximum exponential growth rate in this replicate: within 36% of the mean
(3) Determine the datasets with a maximum exponential growth rate outside of this range
(4) Of these datasets outside the range, remove the dataset with the maximum exponential growth rate furthest from the mean
(5) Re-calculate the mean for the replicate
(6) Repeat steps 2 – 5 until no further datasets are excluded

After 4 iterations, no further datasets were removed. This iterative process was not needed for the determination of the cutoff as that explored the total variation (i.e. the number outside a range) and so would give the same results on which strain to remove, whether iteratively calculating the mean each time a dataset was removed or not.

Data cleaning 2 and calculate log reductions due to dehydration

Those strains with a maximum exponential growth rate outside the range decided in the above analysis of maximum exponential growth rate variation (μ_m needs to be relatively constant over the replicate in order to assume the linear model between inoculum and time to first peak *tmax*).

CODE: 3_ clean_exp_logred.R

(a) Removes those strains with maximum exponential growth rate outside the range
(b) Plots the filtered plots: shows the underlying data, the first peak in the heat flow data and any “odd” characteristics of the curve (e.g. double peaks or exponential growth rate)
(c) fits the linear model using the “fit_line_model.R” (for more information see below)

Section E - Linear model fit and data cleaning 3&4

A linear model is fit to the inoculum and *tmax* data for each strain. The resulting coefficients of this linear model are used to predict the surviving number of bacteria (or inoculum) after the dehydration period based on the extracted *tmax*. This uses the *lm()* function in *R*. The R^2 fit of this to the data is used as an indicator of goodness of fit.

CODE: function_linear_model.R Contains *fit_line_model* function to fit to the data.

For some strains, there are only two datasets pre-dehydration and hence there is only one option for the linear fit. Examples of the fit to data are shown in the below Figure S5. These are outputted by *fit_line_model* to the folder output_fit.

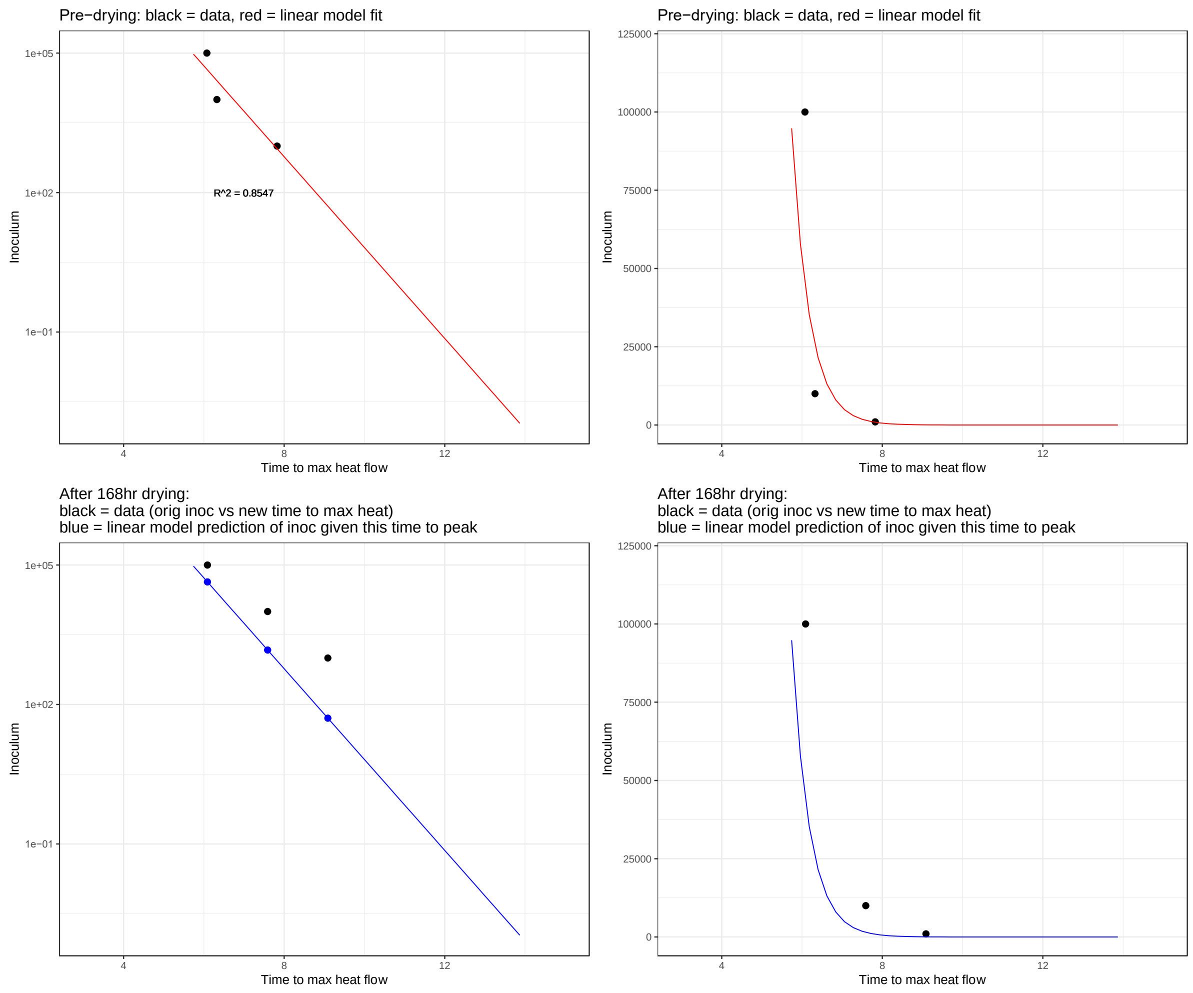

Figure S5. Example of linear fit to pre-dehydration data (red, top) and prediction (blue, bottom) for strain 11090, replicate 8.1. Log scale (left) and linear scale (right)

The range in R^2 values across the replicates is shown in Figure S6. We removed strains with only 2 values pre-dehydration (data cleaning 3). Then we chose a cutoff of 0.75 (data cleaning 4). A summary of remaining data after these cleaning steps is shown in Table S2.

Number of datasets	3	4	5	6	8	9	10	11	12	13	14	15	16	17	18
Number of strains	3	1	5	4	4	2	3	7	8	1	5	13	2	15	25

***Table S2**. The remaining data for analysis after filtering on maximum exponential growth rate, 3 values pre-dehydration and $R^2$with a total of 1330 datasets for 98 strains*

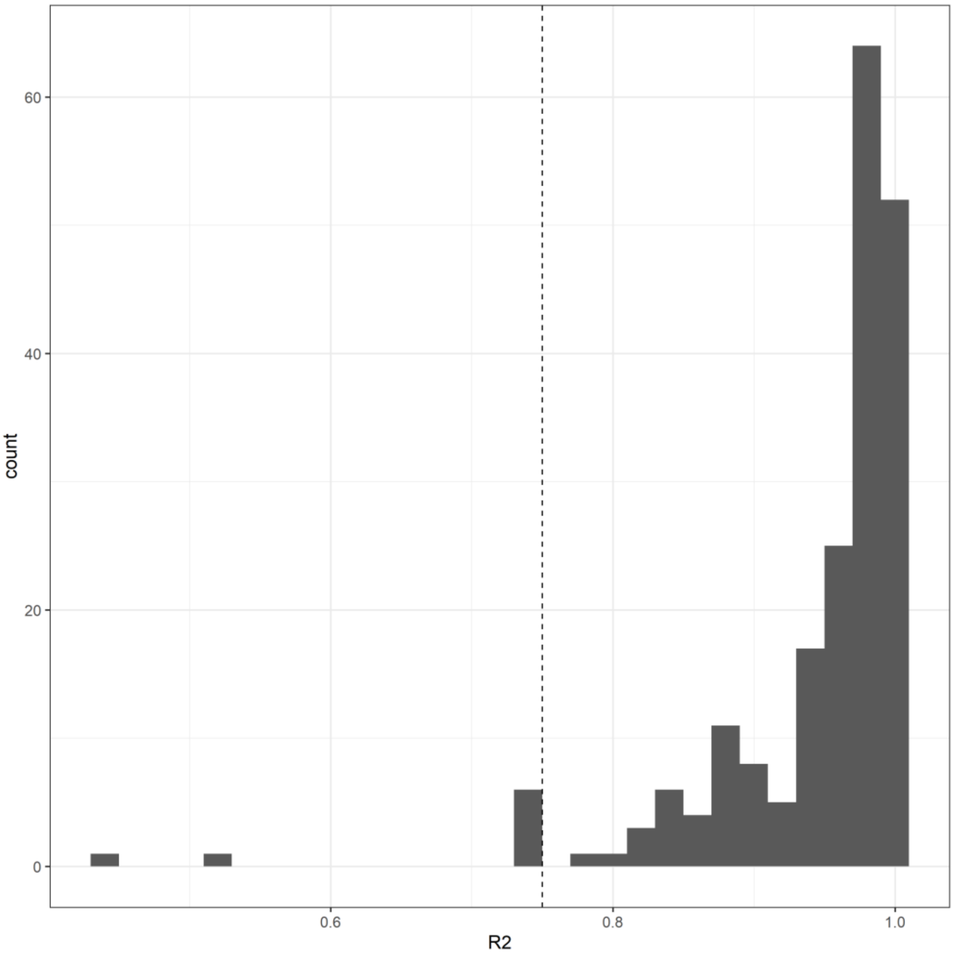

Figure S6. Range in R^2 values for the linear model fit to inoculum vs time to first peak. We used a threshold for the R^2 values of 0.75 (the dashed line).

Supplemental figures

(1) There is a linear relationship between inoculum and time to first peak across all strains

Looking at the individual data for each strain included in the final analysis, a clear linear trend can be seen between inoculum and time to maximum heat flow (Figure S7). It can also be seen that the time to first peak occurs earlier before dehydration than after dehydration (red points to left of blue ones). This difference captures the change in bacterial survival: maximum heat flow is reached at a later time point due to fewer bacteria.

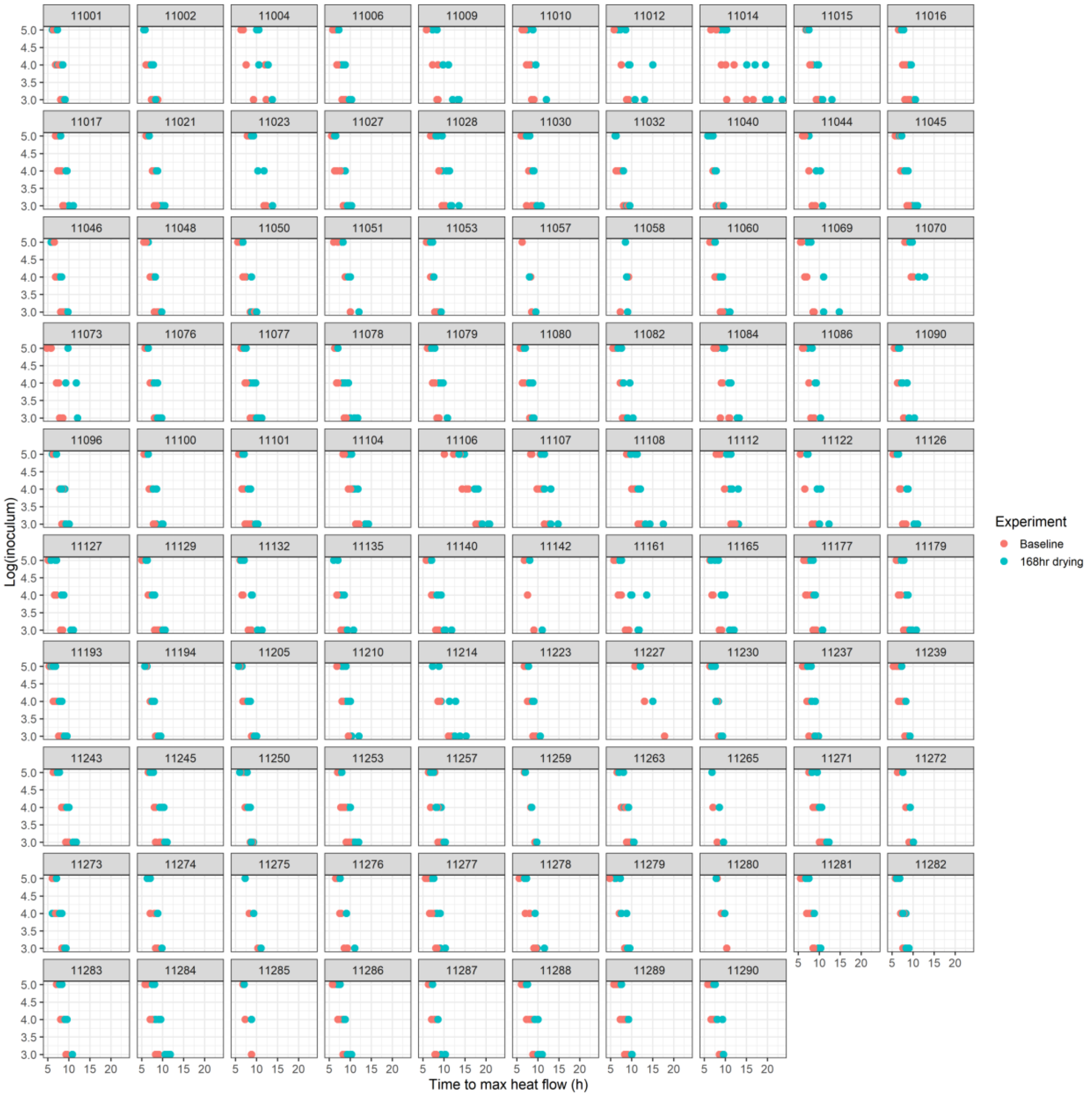

Figure S7: Linear trend for time to first peak (*tmax*) in heat flow against inoculum. Relationship before dehydration is given in red, after dehydration given in blue.

(2) There is some variation in log reduction by strain

The log reduction across replicates is shown below.

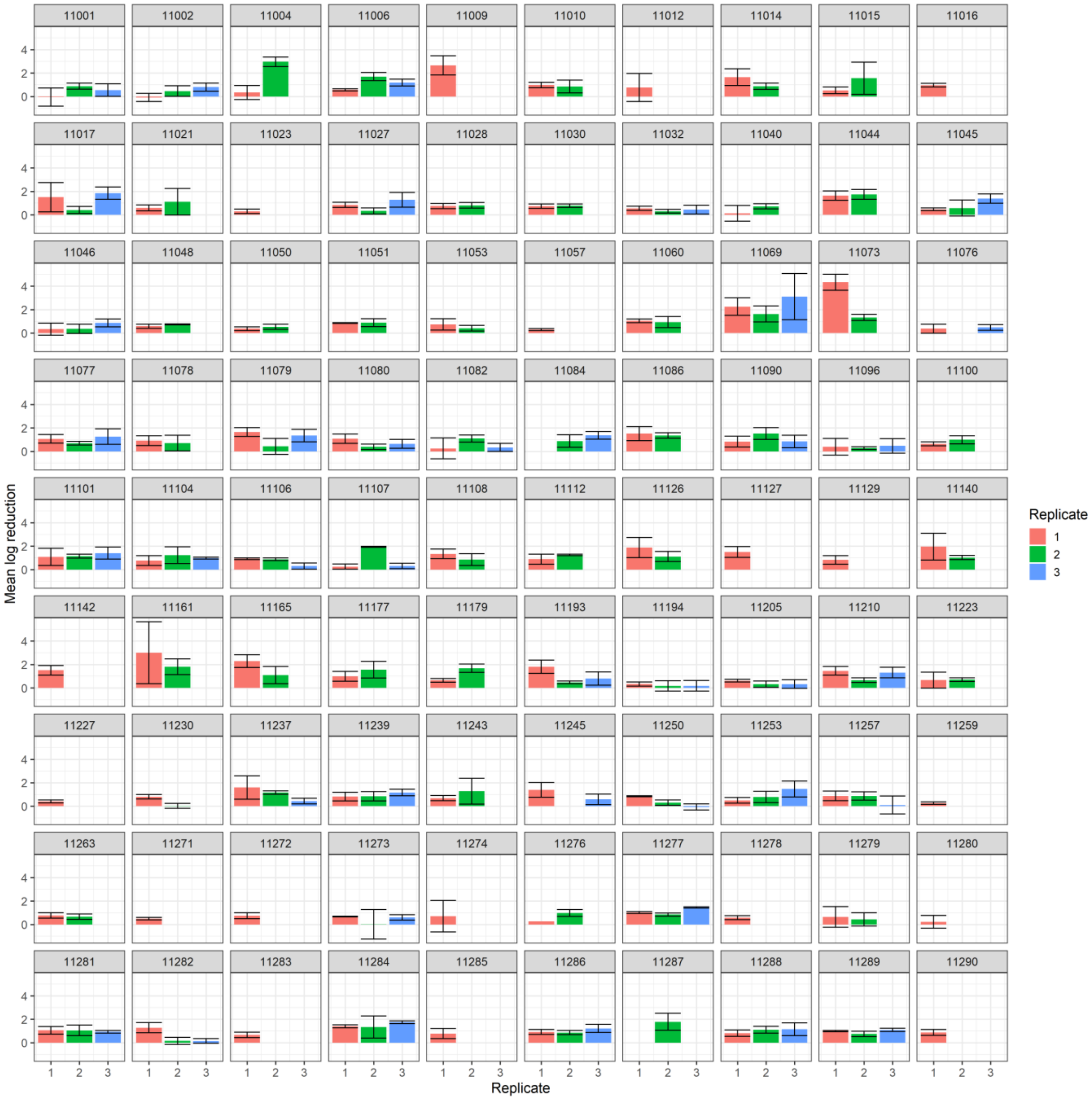

Figure S8: Log reduction across replicates is shown, with error bars being standard deviation over inocula.

(3) The raw data underlying the comparison between country / lineage / success / inoculum

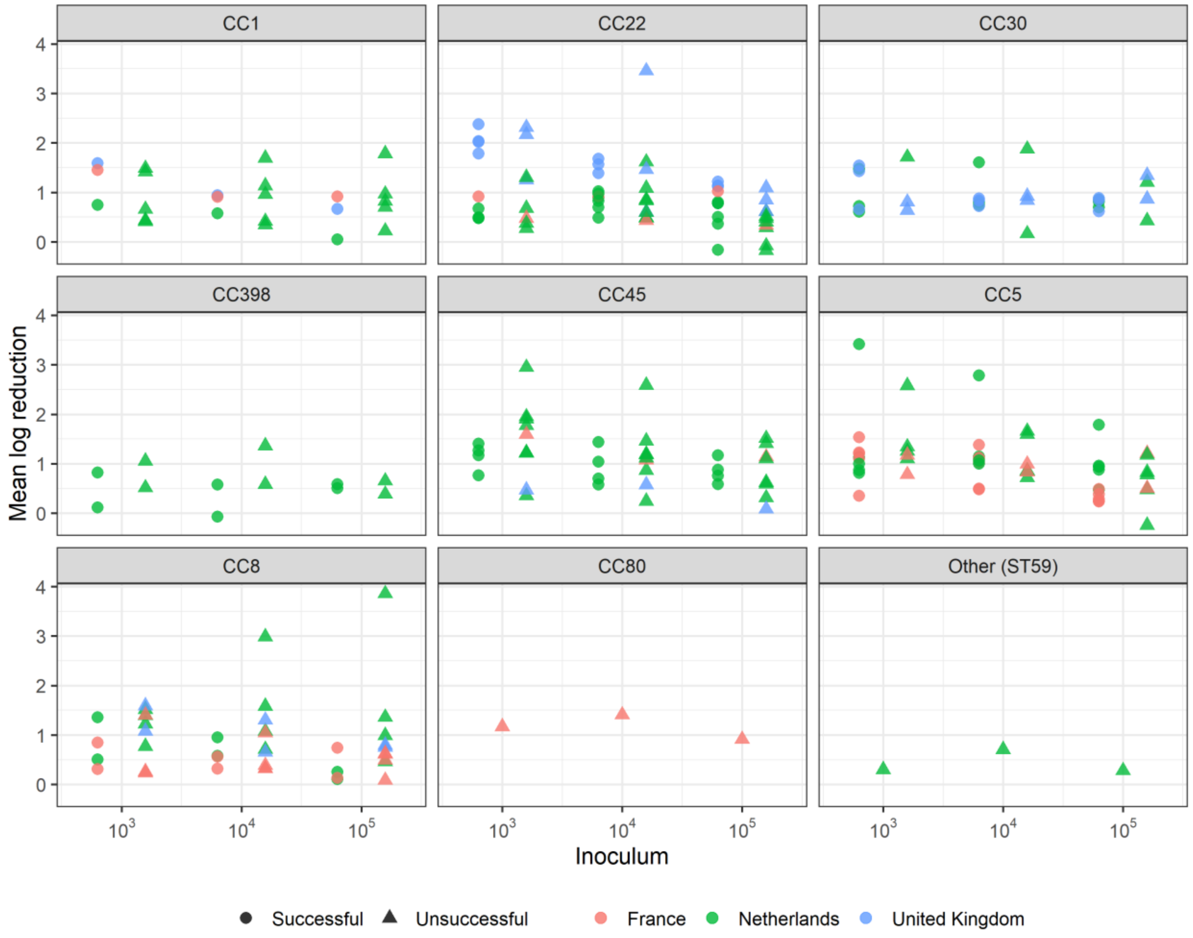

Figure S9. Mean log reduction by starting inoculum (x-axis) for each lineage by country (color) and success (shape). Each point is the mean over the replicates for a single strain.
